# Changes in Hematologic Lab Measures Observed in Patients with Paroxysmal Nocturnal Hemoglobinuria Treated with C5 Inhibitors, Ravulizumab and Eculizumab: Real-World Evidence from a US Based EMR Network

**DOI:** 10.3390/hematolrep15020027

**Published:** 2023-04-21

**Authors:** Jesse Fishman, Seth Kuranz, Michael M. Yeh, Kaylen Brzozowski, Herman Chen

**Affiliations:** 1 Apellis Pharmaceuticals, Inc., Waltham, MA 02451, USA; 2 TriNetX, LLC, Cambridge, MA 02140, USA

**Keywords:** paroxysmal nocturnal hemoglobinuria, C5 inhibitors, eculizumab, ravulizumab, hematological outcome, hemoglobin, anemia, transfusion, breakthrough hemolysis

## Abstract

Paroxysmal nocturnal hemoglobinuria (PNH), a rare acquired hematologic disorder, can be treated with C5 inhibitors (C5i) such as eculizumab or ravulizumab. This retrospective study is the first to describe real-world treatment patterns and changes in hematologic PNH-monitoring laboratory tests among C5i-treated US patients. Data were extracted from TriNetX Dataworks Network and included patients with a PNH diagnosis between 1 January 2010, and 20 August 2021. Patients were stratified into three cohorts based on their C5i usage: eculizumab, ravulizumab (prior eculizumab), and ravulizumab (eculizumab naïve). Hematological markers (hemoglobin [Hb], lactate dehydrogenase [LDH], and absolute reticulocyte count [ARC]) and relevant clinical events (e.g., breakthrough hemolysis [BTH], complement-amplifying conditions [CAC], thrombosis, infection, and all-cause mortality) were captured any time within 12 months post-index treatment. Of the 143 (eculizumab), 43 (ravulizumab, prior eculizumab), and 33 (ravulizumab, eculizumab naïve) patients, mean age across cohorts was 42–51 years, 55–61% were female, 63–73% were White, and 33–40% had aplastic anemia. Among all cohorts 12 months post-C5i treatment, 50–82% remained anemic, 8–32% required ≥1 transfusion, and 13–59% had BTH, of which 33%-54% had CACs. Additionally, thrombosis was seen in 7–15% of patients, infection in 20–25%, and mortality in 1–7%. These findings suggest many C5i-treated patients experience suboptimal disease control.

## 1. Introduction

Paroxysmal nocturnal hemoglobinuria (PNH) is a rare and potentially life-threatening disease characterized by hemolytic anemia, thrombosis, and impaired bone marrow function. This disorder is caused by an acquired somatic mutation in the PIG-A gene on hematopoietic stem cells, which leads to a deficiency in the glycosylphosphatidylinositol (GPI)-anchored proteins, resulting in complement-mediated intravascular and extravascular hemolysis [1]. GPI proteins help integrate complement-inhibitor proteins CD55 and CD59 into the cell membrane, and their surface expression protects hemolysis through blocking the complement cascade and membrane attack complex (MAC). Accordingly, the deficiency or absence of CD55 and CD59 in red blood cells results in chronic-mediated hemolysis in patients with PNH [2]. 

PNH commonly occurs in combination with aplastic anemia and other bone marrow disorders [3]. Frequent patient-reported symptoms include fatigue, headaches, abdominal pain, back pain, excessive weakness, and discomfort from frequent blood transfusions [4]. Treatment for PNH is based on disease severity and may initially involve supportive care, such as blood transfusions, corticosteroids, anticoagulants, and supplementations. However, with disease progression, complement inhibitor medications are often indicated to block the complement cascade and prevent MAC formation and hemolysis [5]. PNH hematological treatment response is assessed by hemoglobin (Hb), lactate dehydrogenase (LDH), absolute reticulocyte count (ARC), and transfusions, and an inadequate response is thought to be tied to the extent of breakthrough hemolysis (BTH) [6]. 

In 2007, eculizumab (ECU) was the first approved humanized monoclonal antibody to target the terminal complement protein C5 and reduce intravascular hemolysis in patients with PNH [7]. Since then, C5 inhibitors (C5i) ECU and subsequently ravulizumab (RAV), which was approved in 2018, have led to improved management and outcomes for patients with PNH, as measured by decreased transfusion dependence and Hb stabilization [7,8,9,10,11]. The differences between eculizumab and ravulizumab lie in their dosing regimens and half-life duration. Specifically, ravulizumab requires less frequent dosing than eculizumab and has a longer half-life, which may improve patient convenience and treatment adherence [10]. In the absence of complement inhibitor treatments, the median survival for patients with PNH following diagnosis is less than 15 years, with thrombosis and bone marrow failure being the leading causes of mortality [12].

Despite C5is significantly reducing hemolysis for many patients with PNH, long-term follow-up studies on patients treated with ECU have found that 18% to 34% continue to require transfusions, and 72% remain anemic [9,13,14]. Additionally, phase 3 trials of C5i have only reported the number of patients with Hb stabilization [7,10,11], whereas international guidance recommends assessing PNH treatment response with Hb normalization (i.e., Hb levels ≥ 12 g/dL) [15]. Thus, although PNH clinical trials have previously reported patient data, the current literature lacks real-world data that reports the recommended hematological parameters and clinical outcomes among patients treated with C5is, especially with the newest C5i, RAV. 

To our knowledge, no studies have reported real-world hematologic measures in the US coincident with C5i treatments. Accordingly, to address these gaps in the literature, we used a real-world electronic medical record (EMR) network to evaluate hemoglobin response, the use of transfusions, BTH events, and long-term clinical outcomes from baseline to 12 months after initiating therapy with ECU or RAV. Our study aimed to follow US patients with PNH for 12 months post-C5i index treatment and report their hematological and clinical outcomes. 

## 2. Methods

### 2.1. Study Design

This retrospective observational study used a treatment decision design that identified cohorts based on when treatment was administered [16]. Given that this is an ultra-rare disease (<20 per million), very few patients were expected to be available for analysis; as such, a case-series cohort design was selected [17,18]. Our case-series design precluded the need for a specific sample size necessary to draw conclusions about the magnitude of the effects. Further, as it is descriptive, it did not require a control group [19]. Data were obtained from the TriNetX Dataworks USA Network, a global federated network of de-identified aggregate EMR data from 44 healthcare organizations representing hospitals, primary care, and specialty providers designed to facilitate research [20,21]. During the current study, the network included data from 55 million patients. TriNetX is described in detail in the Supplement.

The study followed both the International Society of Pharmacoepidemiology Guidelines for Good Pharmacoepidemiology Practice and the Strengthening of the Reporting of Observational Studies in Epidemiology statement [22,23]. Additionally, it was performed per ethical principles consistent with the Declaration of Helsinki, the International Conference on Harmonization Good Clinical Practice guidelines, and the applicable legislation on non-interventional and/or observational studies. Since this study used only de-identified patient records and did not involve the collection, use, or transmittal of individually identifiable data, this study was not required to undergo Institutional Review Board approval.

### 2.2. Patient Eligibility and Stratification

**Figure 1:**
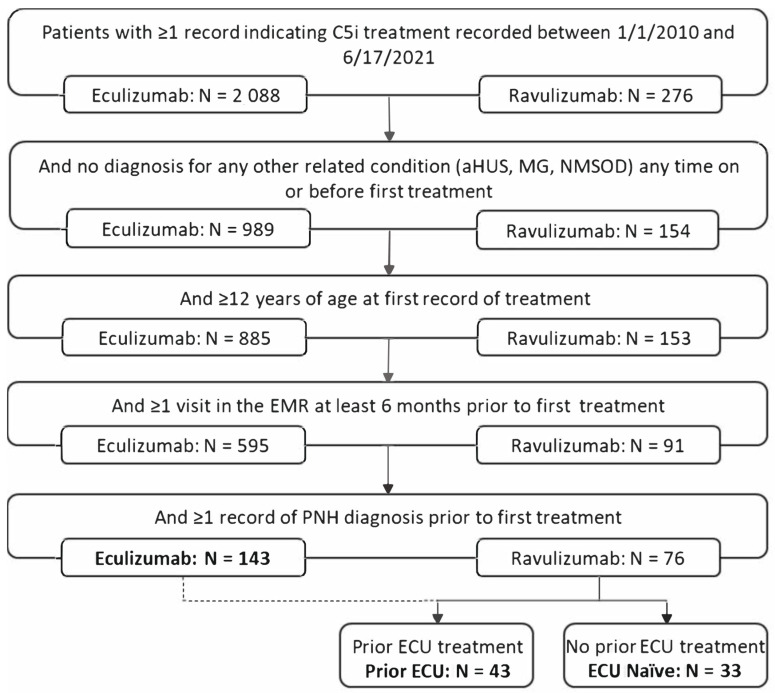
Patient selection. Abbreviations: aHUS, atypical hemolytic uremic syndrome; C5i. C5 inhibitor; EMR, electronic medical record; ECU, eculizumab; MG, myasthenia gravis; NMSOD, neuromyelitis optica spectrum disorder; PNH, paroxysmal nocturnal hemoglobinuria.

All patients aged 12 and older treated with an index C5i between 1 January 2010 and 17 June 2021 and who had a PNH diagnosis before the index date were eligible for inclusion. A PNH diagnosis was identified based on the International Classification of Diseases 9th or 10th Revision Clinical Modification codes 283.2 and D59.5, respectively. Additionally, patients without at least one medical visit recorded six months or more before the index date (to identify patients with a history of care within the healthcare organizations and who were likely to have baseline laboratory values recorded), and those with a prior history of myasthenia gravis, atypical hemolytic uremic syndrome, or neuromyelitis optica spectrum disorder were excluded (Figure 1). The baseline period comprised the 12 months before and including the index date.

Patients were stratified into three cohorts by C5i treatment history which defined their exposure: ECU, RAV (ECU naïve), and RAV (prior ECU). ECU patients who switched to RAV were included in either the ECU cohort or the RAV (prior ECU) cohort, depending on the analyzed time-window. The ECU population was not further segmented because no patients switched from RAV to ECU. C5i treatment was identified by medication or procedure records in the EMR. Hence, as this study did not utilize prescription claims, it was not necessary to use specific time limits or predefined gaps between each of the C5i administration records.

Patient demographics, including age at index, sex, and race, were captured from the EMR. Comorbid conditions identified in the baseline period included aplastic anemia, anemia (any), arterial or venous embolism, gastroesophageal reflux disorder, hypertension, myelodysplastic syndrome, and thrombocytopenia. 

### 2.3. Study Outcomes

The primary outcomes defined a priori were the proportions of C5i-treated patients with PNH who had hematological responses as measured by changes in Hb and LDH levels from baseline during the observation period. Secondary outcomes included the proportion of patients receiving transfusions, experiencing BTH and complement-amplifying conditions (CACs), the incidence of long-term clinical outcomes (e.g., thrombosis, infection, infusion reaction, persistent anemia, and mortality), and changes in ARC values from baseline during the observation period. These outcomes were assessed at various time points within the 12-month post-index period to reflect what is done in clinical practice and to match hematologic assessments performed in PNH clinical trials [11,24]. 

Hb, LDH, and ARC lab values were captured in four-time windows: baseline, 1–3 months, 4–6 months, and 7–12 months post-index. The distribution of the number of laboratory measures over the four-time windows can be found in Table S1. Hb values greater than 20 g/dL and LDH and ARC values more than three times above the standard deviation (i.e., LDH values greater than 2190 U/L or ARC values greater than 17,200 × 10^9^/L) were removed based on clinical feasibility. The baseline value was the lab value closest to the index date within the baseline period. The values in the follow-up windows in the Hb, LDH, and ARC-specific analyses were those recorded closest to the end of the time period. For LDH, we captured those with LDH levels greater than 360 U/L, or 1.5 times the upper limit of normal (ULN). 

Further, those with Hb stabilization, response, or normalization were captured in two time windows: 4–6 months and 7–12 months. PNH clinical trial literature defined Hb stabilization as no measured decrease in Hb levels ≥ 1 or ≥2 g/dL from baseline; Hb response as an increase in Hb levels ≥ 1 g/dL from baseline; and Hb normalization as achieving Hb levels ≥ 12 g/dL [7,10,11]. The Hb analysis only included patients who did not receive a transfusion in the 31–365 days post-index period and had at least one Hb lab result in the baseline period, which allowed for a 30-day treatment adjustment period and reduced the confounding effect of transfusion on Hb improvement.

Three definitions of BTH using PNH expert consensus from the literature were used for analysis [25]:

1. LDH of at least 480 U/L (≥2 × ULN) and at least one new symptom or sign of intravascular hemolysis (i.e., fatigue, hemoglobinuria, abdominal pain, dyspnea, anemia (Hb < 10 g/dL), major adverse vascular event (including thrombosis), dysphagia, or erectile dysfunction) within one, three, or seven days of the elevated LDH.
2. LDH of at least 480 U/L (≥2 × ULN) alone, regardless of other signs/symptoms.
3. Elevated LDH (≥50% increase from baseline) and decreased Hb (Hb ≥ 2 g/dL from baseline) within one week of one another and measured within ≥4 months after index.

Given the lack of clinically available BTH data in patients with PNH following a treatment switch in prior literature, our analysis focused on identifying BTH events within each C5i group and following a treatment switch from ECU to RAV. Definitions 1 and 2 were analyzed for those with at least one LDH lab result after six or 12 months of continuous C5i treatment. Definition 3 was analyzed after four months of continuous C5i treatment for patients with at least one LDH and one Hb lab result in both the baseline period and after four months post-index. Time frames for analyses were drawn from clinical trials and common clinical practice. Additionally, as CACs (e.g., pregnancy, infection, major surgery) increase the risk of BTH, we also assessed for CACs if they occurred within 15 days before or after the patients’ first BTH event in the relevant time frame, as defined by PNH expert definitions [26].

### 2.4. Follow-up and Event Rates

A complete case approach for this study included ensuring treatment with ECU or RAV was for the specific PNH indication and duration as outlined per study inclusion and cohort definitions. However, as patients within each cohort had variable follow-up times, our analyses calculated and reported person-time rates from all observed data as per best practices in cohort study design [27,28]. The follow-up period included the day after the index date through the earliest of the following: date of death, end of the patient record, date of discontinuation, or August 20, 2021. The date of discontinuation was defined as the day of the last C5i treatment plus 14 days for ECU or 56 days for RAV. The full study schema is provided in Figure S1A. 

### 2.5. Treatment Switch

This analysis used a two-stage model that allowed for the first appearing administration date of the new treatment to be used to identify a treatment switch. The methodological basis for the two-stage model for use in observational research has been described extensively and validated for use in submissions to the Food and Drug Administration (FDA) and National Institute for Healthcare Excellence (NICE) [29,30]. In this analysis, the two stages would occur when a patient appeared in both the ECU cohort and the RAV (prior ECU) cohort, in which case the patient would have two index dates: one at the start of ECU treatment and one at the start of RAV treatment. They would have two non-overlapping follow-up periods as the follow-up period for ECU ended at the earliest of either the discontinuation of ECU or the initiation of RAV. However, the baseline period for the RAV (prior ECU) treatment could include all or some of the follow-up period for their ECU treatment and some of their baseline period for their ECU treatment. For patients in both cohorts, their hematologic parameters were considered as baseline values at the time of their respective index dates and as follow-up values during the time of the respective treatments to account for any exposure-response relationship. An example of how such a patient would be identified and included in each cohort appears in Figure S1B. 

### 2.6. Statistical Analysis

Categorical data were analyzed using descriptive statistics and summarized using frequency counts and percentages. Continuous data were also analyzed descriptively and summarized using mean, median, standard deviation (SD), and interquartile range. Patients with missing laboratory information during a given time frame of interest did not contribute to the denominators of categorical measures or the summary measures of continuous data for the said time frame. All results were analyzed overall and by subgroup based on the initial PNH treatment group classification. Due to the small sample sizes, significance testing was not conducted for the analyses, and instead, descriptive analyses were used for comparisons. All analyses were performed in SAS version 9.4. 

## 3. Results

Overall, 176 PNH patients treated with C5i met the study inclusion criteria (Figure 1). Among these, 143 (81.3%) received ECU, and 76 (43.2%) received RAV. Among those who received RAV, 33 (43.4%) were naïve to ECU, and 43 (56.6%) patients had prior ECU treatment. 

The mean age of patients with PNH at baseline ranged from 42.6 years to 51.0 years (Table 1). Patients below the age of 18 were not present in the RAV analysis cohorts and only represented 3.5% of the patients in the ECU cohort. Most patients in all three analysis cohorts were female (55.2–60.5%), White (62.8–72.7%), and in the 35–64 age group (49.0–60.6%).

More than 60% of patients in any cohort had some form of anemia, and aplastic anemia was documented at similar rates across the three groups (33–40%). Myelodysplastic syndrome was uncommon (<8%) in all cohorts.

### 3.1. Hemoglobin

**Figure 2:**
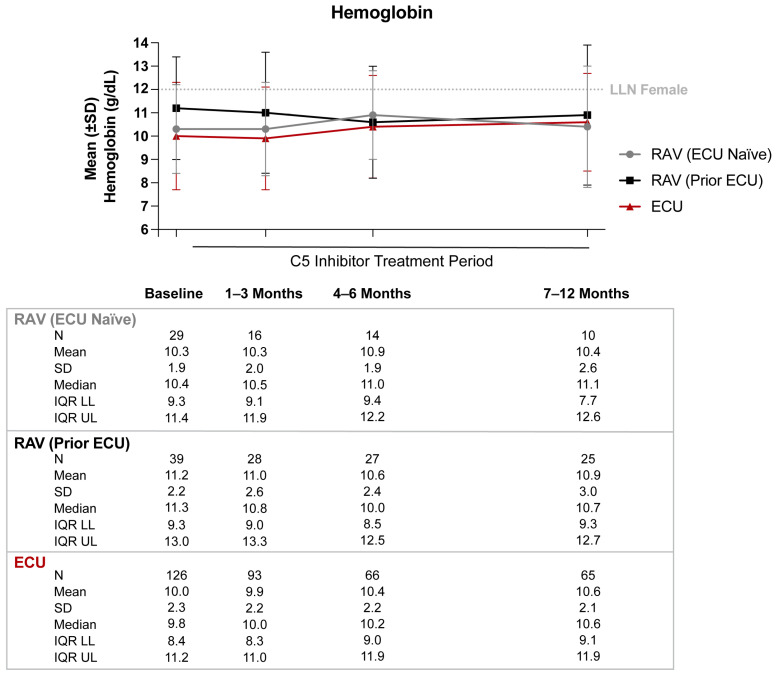
Hemoglobin levels from baseline through 12 months of C5 inhibitor (C5i) treatment. Abbreviations: C5i, C5 inhibitor; ECU, eculizumab; IQR LL, interquartile range lower limit; IQR UL, interquartile range upper limit; LLN, lower limit of normal; RAV, ravulizumab; SD, standard deviation. Note: Mean (±SD) hemoglobin levels were graphed. The LLN for hemoglobin was defined as 12 g/dL.

No considerable improvements in mean Hb levels at 12 months were observed in the RAV population, irrespective of prior treatment status (Figure 2). ECU-treated patients displayed modest improvements in mean Hb levels after 12 months of treatment (mean (SD) Hb baseline, 10.0 (2.3) g/dL; mean (SD) Hb 7–12 months, 10.6 (2.1) g/dL). However, mean Hb levels in all three cohorts remained below the lower limit of normal through 12 months of C5i treatment. 

Among all patients, 8.0–32.3% had at least one transfusion during the 12 months post-index (Table 2). Stabilization of Hb levels within 2 g/dL was observed in 50.0–68.0% of C5i-treated patients at 12 months; however, approximately 70% of C5i-treated patients failed to have a Hb response (≥1 g/dL increase in Hb levels from baseline), and 64.3–77.8% and 50.0–82.3% of C5i-treated patients failed to have Hb normalization (Hb levels ≥ 12 g/dL) at six and 12 months, respectively.

### 3.2. Lactate Dehydrogenase

**Figure 3:**
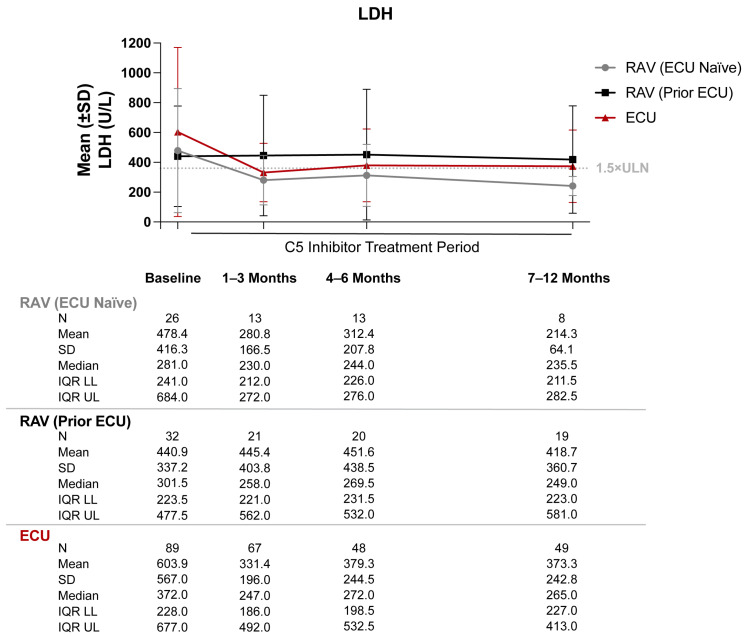
Lactate dehydrogenase (LDH) levels from baseline through 12 months of C5 inhibitor (C5i) treatment. Abbreviations: ECU, eculizumab; IQR LL, interquartile range lower limit; IQR UL, interquartile range upper limit; RAV, ravulizumab; SD, standard deviation; ULN, upper limit of normal. Notes: Mean (±SD) LDH levels were graphed. 1.5 times the ULN for LDH was defined as 360 U/L.

**Figure 4:**
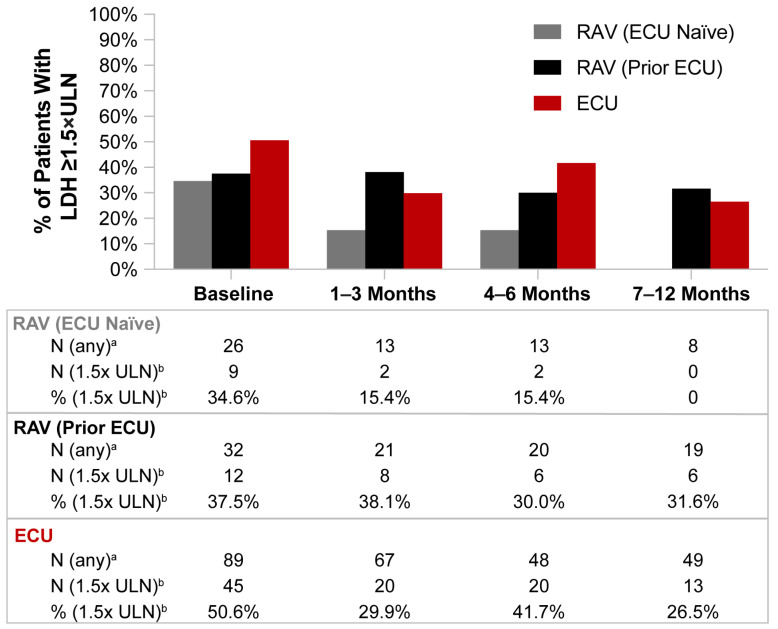
Lactate dehydrogenase (LDH) levels ≥ 1.5 × the upper limit of normal (ULN) from baseline through 12 months of C5 inhibitor (C5i) treatment. Abbreviations: ECU, eculizumab; LDH, lactate dehydrogenase; RAV, ravulizumab; ULN, upper limit normal. Notes: 1.5 times the ULN for LDH was defined as 360 U/L. Due to the small sample sizes, significance testing was not conducted for the analyses, and descriptive analyses were used for comparisons. For a more detailed analysis of LDH, refer to the BTH analysis in Table 3. ^a^ Number of patients with at least one test during the time period of interest. ^b^ Number (%) of patients with at least one test result 1.5 times above the ULN.

Treatment naïve patients in the ECU and RAV groups had some decrease in mean LDH levels from baseline to 12 months (603.9 U/L to 373.3 U/L for ECU; 478.4 U/L to 241.3 U/L for RAV, ECU naïve) (Figure 3). By comparison, RAV-treated patients with prior ECU experience had minimal to no decrease from baseline to 12 months (440.9 U/L to 418.7 U/L). At 12 months, LDH levels remained greater than or equal to 1.5 times the upper limit of normal (360 U/L) in 0%, 31.6%, and 26.5% of tested patients treated with RAV (ECU naïve), RAV (prior ECU), and ECU patients, respectively (Figure 4). 

### 3.3. Breakthrough Hemolysis and Complement-Amplifying Conditions

Based on expert consensus definitions, when assessing BTH among patients in the ECU and overall RAV groups, 41 ECU-treated and 23 RAV-treated patients were eligible for analyses based on definitions 1 and 2, and 44 ECU-treated and 32 RAV-treated patients for analysis based on definition 3. As per definition 1 of new signs or symptoms of hemolysis ±7 days relative to an elevated LDH value after more than 12 months of treatment, 10 (24.4%) ECU patients and one (4.3%) RAV patient had BTH (Table 3). When the definition was expanded to include all patients with elevated LDH alone regardless of other signs/symptoms after 12 months of treatment, 24 (58.5%) ECU patients and three (13.0%) RAV patients had BTH as per definition 2. Regarding definition 3 of a concurrent increase in LDH and decrease in Hb after more than four months of treatment, BTH was seen in 12 (27.3%) ECU patients and three (9.4%) RAV patients. Of note, among those who experienced BTH (as defined by any of the three criteria), 54.2–78.6% of the ECU-treated patients and 33.3–100% of RAV-treated patients had a CAC within 15 days before or after the BTH event (Table 4).

Further, when assessing BTH following a treatment switch from ECU to RAV, 18 patients in the RAV (prior ECU) were included in analyses for definitions 1 and 2, and 20 RAV (prior ECU) patients were included for definition 3, of which one (5.6%) patient in the RAV (prior ECU) group had BTH using definition 1, three (16.7%) when using definition 2, and two (10.0%) when using definition 3 (Table 3).

### 3.4. Absolute Reticulocyte Count

Among the PNH monitoring labs, ARC was the least commonly reported, with at most 16.3% of patients in any cohort having a test result in the follow-up period (Figure S2). Among patients receiving ECU and RAV (prior ECU), ARC values rose from a mean of 79.2 × 10^9^/L and 94.5 × 10^9^/L, respectively, at baseline to 113.7 × 10^9^/L and 135.9 × 10^9^/L at 12 months. ARC trends could not be determined in RAV (ECU naïve) patients due to the low count of patients with a test result (<5 patients in any follow-up window). 

### 3.5. Long-Term Clinical Outcomes 

The incidence of long-term clinical outcomes, including thrombosis, kidney disease, and mortality, differed between C5i-treatment groups (Table 5). Only events which occurred at least 30 days following the index treatment through the end of the treatment period were analyzed. The number (proportions) of patients who had venous or arterial thrombosis or embolism on RAV was 10 (13.2%), and on ECU was 22 (15.4%). Additionally, seven (9.2%) RAV-treated patients and 27 (18.9%) ECU-treated patients had kidney disease as defined as a diagnosis of chronic kidney disease, kidney failure, dependence on renal dialysis, or nephritis. Over the study period, there were 11 deaths in the C5i-treated groups (RAV, *n* = 1 (1.3%); ECU, *n* = 10 (7.0%)). Of note, the proportion of patients with persistent anemia, infusion reactions, and infections appeared similar among the groups. Persistent anemia, defined as Hb < 10g/dL for 8 weeks, was seen in nearly one in four patients in both treatment groups (RAV, 22.4% and ECU, 26.6%). Approximately 20% of all patients treated with RAV or ECU had serious infections (i.e., meningococcal infection, sepsis, aspergillus infection, viral infection, respiratory tract infection, urinary tract infection, cellulitis, pneumonia, and viral gastroenteritis) and infusion reactions. Further, of the patients who had a clinical outcome at least 30 days following the index treatment, 53.3–100.0% of ECU-treated patients and 29.4%–100.0% of RAV-treated patients had at least two instances of the outcome recorded at least 30 days apart.

## 4. Discussion

To our knowledge, this is the first study to describe real-world C5i treatment patterns in the US and changes in hematologic PNH-monitoring laboratory tests among C5i-treated patients. Existing real-world data on C5i efficacy come predominantly from single-site studies and registry data of ECU-treated patients primarily outside of the US. Our study used an EMR network to follow US patients with PNH for 12 months post C5i-index treatment to report hematological response, transfusions and BTH events, and long-term clinical outcomes from baseline to 12 months after initiating therapy with ECU or RAV. 

In this study, patients with PNH receiving C5i therapy with ECU or RAV displayed minimal long-term improvements in hematologic outcomes. Although Hb levels did not substantially worsen in patients treated with C5 inhibitors, 50–82% of patients remained anemic at 12 months post-index, with Hb levels below the lower limit of normal (12 g/dL). In fact, 52–76% of C5i-treated patients failed to have Hb stabilization within 1 g/dL and 70–72% failed to have Hb response of ≥1 g/dL increase from baseline. Additionally, 32% of RAV-treated (prior ECU) and 27% of ECU-treated PNH patients had LDH levels greater than or equal to 1.5 times the upper limit of normal (360 U/L) from baseline through 12 months following initiation of C5i treatment. The mean ARC in PNH patients receiving RAV (prior ECU) also remained 1.5 times above the upper limit of normal (120 × 10^9^/L) despite treatment. 

Further, many PNH patients treated with ECU or RAV continued to require blood transfusions and had poor long-term clinical outcomes. Over the study period, 8–32% of C5i-treated patients received at least one transfusion, and 13%-59% had BTH (defined as an elevated LDH over twice the upper limit of normal (480 U/L) regardless of signs and symptoms), of which 33–54% also had a CAC within 15 days of BTH. In addition to persistent anemia, the long-term clinical outcomes with the highest incidence were infusion reaction, infection, and thrombosis. 

This analysis supports evidence from existing C5i clinical trials in that there may be an incomplete response to C5i treatment, primarily through using hematological parameters and secondarily through the proportion of patients requiring transfusions, experiencing BTH and long-term clinical complications. In the phase 3 trial of ECU, 51% of patients did not have Hb levels above 7.7 g/dL, and 49% of patients required at least one transfusion during the six-month follow-up. In addition, mean LDH levels dropped significantly upon initiation of treatment but remained above the upper limit of normal throughout the follow-up period [7]. Similarly, in a phase 3 trial of RAV versus ECU in C5i naïve patients, more than 50% in both cohorts had LDH levels above the upper limit of normal at six months, while 26.4% of RAV and 33.9% of ECU-treated patients required a transfusion [11]. Approximately one-third of patients treated with RAV (32.0%) and ECU (35.5%) did not have Hb stabilization, defined as a decrease of no more than 2 g/dL. Our study was not able to corroborate clinical trial findings of no BTH following initiation of RAV among patients who switched from ECU [10].

Furthermore, three other studies using either single-site data or registries yielded similar results. In a study of 56 patients at the University Hospital Essen, Germany, mean Hb and LDH values before treatment initiation were 9.7 g/dL and 1480 U/L, respectively [31]. In months 7–12 after starting ECU, 19.2% required a transfusion, 76.3% had Hb less than 12 g/dL, 16.3% had LDH values greater than 1.5 times the upper limit of normal, and 36.1% had ARC values greater than 1.5 times the upper limit of normal. Trends were similar during any six months over a mean of 5.24 years of follow-up. An analysis of 79 PNH patients treated with ECU in the Leeds center of the UK PNH National Service found that median LDH values dropped from 2,872 U/L to 477 U/L but remained above the ULN for most patients [9]. In addition, 34% of patients required transfusions within 12 months of treatment initiation. Lastly, an analysis of 779 PNH patients treated with ECU from the International PNH Registry found that mean Hb lab values at the most recent follow-up ranged from 10.1 to 10.7 g/dL, depending on the presence of comorbid aplastic anemia, and LDH values ranged from 1.2–1.4 times the upper limit of normal, while ARC values ranged from 162.5–191.2 × 10^9^/L [32].

Altogether, these descriptive results demonstrate that anemia is still persistent in this C5i-treated cohort. A likely explanation is the presence of incomplete inhibition of both intravascular hemolysis, as suggested by high LDH levels on therapy, and extravascular hemolysis, which is unaddressed by C5 inhibition. In fact, molecular studies on red blood cells of ECU and non-ECU-treated patients have demonstrated that extravascular hemolysis occurred through a C3-mediated pathway and that PNH RBCs are susceptible to C3 binding [33,34]. Interestingly, PNH patients with polymorphisms to the complement receptor 1 gene, seen especially in Asia and Europe, had a higher abundance of hemoglobin with bound C3 and were more likely to be sub-optimal responders to ECU [35]. The study confirmed that by affecting C3 binding, patients with the polymorphism had an improved response to C5i treatments. Accordingly, recent clinical trials of the new C3-inhibitor, pegcetacoplan, have shown improved hematological outcomes in patients with PNH through targeting both hemolytic pathways [24]; however, there were no C3i patient data available at the time this analysis was conducted which should be addressed in a future study.

Although our study confirmed the drug administration of C5i through the verification of procedure codes from the IV administration of C5is, this analysis was unable to determine dosing patterns. The evaluation of dose escalations among patients in the US is an important area of future research as analyses in German and UK single-site studies found C5i treated patients were receiving a higher-than-labeled dose of ECU among 6.3–21.4% of patients [9,14,31]. Our analysis also lacked cost information, as previous analyses of costs associated with PNH showed that many ECU-treated patients continued to experience uncontrolled disease [36]. On average, patients had 0.2 PNH-related inpatient admissions per person-year, costing an average of USD 5651 per person per year [36]. Future analyses should attempt to evaluate the cost implications associated with disease control, as well as compare outcomes between C5i and C3 treatments, such as pegcetacoplan, to inform clinical and economic decision-making for patients with PNH. 

### Limitations

There are several limitations to this study. While EMR databases are useful for analyzing clinical outcomes and treatment trends, one fundamental drawback is that the data are retrospective and observational. Additionally, this analysis is limited as it was descriptive and complex modeling was not used to more fully attribute associations of the observed events reported in this study which may or may not directly be attributed to the drug exposure. Thus, we could not infer causation or draw inferences from the associations observed. Future epidemiological analysis might attempt to assess the associations of C5i treatment and all-cause mortality and other safety events of interest. Further, despite efforts to standardize data input into EMR, data quality and preprocessing issues are inevitable. For example, the data are subject to data entry errors, diagnostic and procedure coding errors, and missing data that may occur as providers interact with EMR systems. Although TriNetX checks for consistency in lab values across contributing healthcare organizations, it does not perform any specific method for data sanitization, allowing individual researchers to independently make these choices [21]. 

This study was also limited by the small sample size due to the rarity of PNH. Given that not all medical records across provider settings were captured, this could have impacted hematologic and disease progression (i.e., unrecorded transfusion and unrecorded comorbidities). Additionally, multiple patients had missing laboratory values within the various timeframes used for these analyses, which may have resulted in underreporting the frequency of BTH. Lab test accuracy and precision also vary by institution and cannot be determined from the data provided to TriNetX. Lastly, TriNetX data predominantly capture large academic medical centers and may not represent patients treated outside the providing healthcare organization.

## 5. Conclusions

Although open-label trial data exist for C5i in the PNH population, this study fills an important gap in the literature regarding real-world hematologic results for C5i-treated PNH patients in the US, where previously there had only been data on ECU captured at single sites or in registries and scant real-world data in the US exist on RAV. In this analysis of long-term clinical outcomes, over 50% of PNH patients treated with C5is did not have a Hb response (Hb ≥ 1 g/dL increase from baseline) or normalization (Hb ≥ 12 g/dL). Accordingly, this study highlights the need for improved therapy for patients with PNH and future research should assess the real-world treatment patterns and hematologic responses of alternative therapies, such as C3-inhibition treatments. 

## Figures and Tables

**Table 1:** Baseline characteristics.

	RAV (ECU Naïve) N = 33				RAV (Prior ECU) N = 43				ECU N = 143			
	n/ mean	%/ SD	LL 95% CI	UL 95% CI	n/ mean	%/ SD	LL 95% CI	UL 95% CI	n/ mean	%/ SD	LL 95% CI	UL 95% CI
Age at index (mean, SD)	51	16.7	45.3	56.7	44.2	15.6	39.5	48.9	42.6	17.2	39.8	45.4
Age group at index (n, %)												
12–17	0	0.0%	-	-	0	0.0%	-	-	5	3.5%	-	-
18–34	7	21.2%	7.3%	35.2%	11	25.6%	12.5%	38.6%	48	33.6%	25.8%	41.3%
35–64	20	60.6%	43.9%	77.3%	26	60.5%	45.9%	75.1%	70	49.0%	40.8%	57.1%
65+	6	18.2%	5.0%	31.3%	6	14.0%	3.6%	24.3%	20	14.0%	8.3%	19.7%
Sex (n, %)												
Female	18	54.5%	37.6%	71.5%	26	60.5%	45.9%	75.1%	79	55.2%	47.1%	63.4%
Male	15	45.5%	28.5%	62.4%	16	37.2%	22.8%	51.7%	64	44.8%	36.6%	52.9%
Race (n, %)												
White	24	72.7%	57.5%	87.9%	27	62.8%	48.3%	77.2%	93	65.0%	57.2%	72.9%
Black or African American	5	15.2%	2.9%	27.4%	6	14.0%	3.6%	24.3%	23	16.1%	10.1%	22.1%
Other ^a^	1	3.0%	-	-	2	4.7%	-	-	5	3.5%	-	-
Unknown	3	9.1%	−0.7%	18.9%	8	18.6%	7.0%	30.2%	22	15.4%	9.5%	21.3%
US region (n, %)												
Northeast	4	12.1%	1.0%	23.3%	7	16.3%	5.2%	27.3%	23	16.1%	10.1%	22.1%
Midwest	5	15.2%	2.9%	27.4%	7	16.3%	5.2%	27.3%	20	14.0%	8.3%	19.7%
South	19	57.6%	40.7%	74.4%	13	30.2%	16.5%	44.0%	62	43.4%	35.2%	51.5%
West	5	15.2%	2.9%	27.4%	15	34.9%	20.6%	49.1%	38	26.6%	19.3%	33.8%
Clinical characteristics ^b^ (n, %)												
Any anemia	20	60.6%	43.9%	77.3%	28	65.1%	50.9%	79.4%	88	61.5%	53.6%	69.5%
Aplastic anemia	11	33.3%	17.2%	49.4%	16	37.2%	22.8%	51.7%	57	39.9%	31.8%	47.9%
Myelodysplastic syndrome	2	6.1%	-	-	1	2.3%	-	-	11	7.7%	-	-
Hypertension	7	21.2%	7.3%	35.2%	7	16.3%	5.2%	27.3%	21	14.7%	8.9%	20.5%
Thrombocytopenia	11	33.3%	17.2%	49.4%	15	34.9%	20.6%	49.1%	47	32.9%	25.2%	40.6%
GERD	4	12.1%	-	-	3	7.0%	-	-	15	10.5%	-	-
Venous embolism	3	9.1%	−0.7%	18.9%	11	25.6%	12.5%	38.6%	20	14.0%	8.3%	19.7%
Arterial embolism	0	0.0%	-	-	0	0.0%	-	-	0	0.0%	-	-
Follow-up time (mean days, SD)												
Total available follow-up time	690	971	358.7	1021.3	420	274	338.1	501.9	224	201	191.1	256.9
Follow-up capped at 12 months	201	161	146.1	255.9	270	133	230.2	309.8	184	135	161.9	206.1
Treatment duration ^c^												
Mean days, SD	642	958	315.1	968.9	382	273	300.4	463.6	184	198	151.5	216.5
Median days, IQR	166	0–931	-	-	404	41–619	-	-	104	27–363	-	-

Abbreviations: C5i, C5 inhibitor; CI, confidence interval; ECU, eculizumab; GERD, gastroesophageal reflux disease; IQR, interquartile range; LL, lower limit; RAV, ravulizumab; SD, standard deviation; UL, upper limit; US, United States. ^a^ “Other” race includes persons of Asian, American Indian, Alaska Native, Native Hawaiian, or other Pacific Islander race. ^b^ Comorbidities identified by diagnosis codes in the baseline period (Table S2). ^c^ Treatment duration was defined as the time from first C5i record to last C5i record, adding 14 days for ECU treatment and 56 days for RAV treatment to account for typical treatment courses. Treatment duration was assessed as the total duration of all C5i treatment among those receiving either C5i; the total duration of ECU treatment among those receiving ECU, stratified by those switching and not switching to RAV; and the total duration of RAV treatment among those receiving RAV, stratified by prior ECU and ECU naïve.

**Table 2:** Hemoglobin (Hb) performance for RAV and ECU-treated patients who did not receive any Hb transfusions.

	RAV (ECU Naïve) N = 33		RAV (Prior ECU) N = 43		ECU N = 143	
Hb performance 91–180 days post-index ^a^						
Patients with ≥1 Hb lab value, N	14		27		63	
Average person-time until last Hb value in the time frame (months), mean (SD)	5.1	0.8	5.0	0.7	5.2	0.8
Patients with ≥1 transfusion, N (%)	2	14.3%	2	7.4%	17	27.0%
Hb stabilization within 2 g/dL, N (%)	10	71.4%	20	74.1%	42	66.7%
Hb stabilization within 1 g/dL, N (%)	7	50.0%	16	59.3%	29	46.0%
Hb response; Hb ≥1 g/dL increase from baseline, N (%)	3	21.4%	5	18.5%	19	30.2%
Hb normalization; Hb ≥12 g/dL, N (%)	5	35.7%	7	25.9%	14	22.2%
Hb performance 181–365 days post-index ^b^						
Patients with ≥1 Hb lab value, N	10		25		62	
Average person-time until last Hb value in the time frame (months), mean (SD)	10.9	1.7	10.6	1.1	10.7	1.6
Patients with ≥1 transfusion, N (%)	3	30.0%	2	8.0%	20	32.3%
Hb stabilization within 2 g/dL, N (%)	5	50.0%	17	68.0%	33	53.2%
Hb stabilization within 1 g/dL, N (%)	3	30.0%	12	48.0%	15	24.2%
Hb response; Hb ≥1 g/dL increase from baseline, N (%)	3	30.0%	7	28.0%	18	29.0%
Hb normalization; Hb ≥12 g/dL, N (%)	5	50.0%	11	44.0%	11	17.7%

Abbreviations: C5i, C5 inhibitor; ECU, eculizumab; Hb, hemoglobin; RAV, ravulizumab; SD, standard deviation. Note: The Hb performance analysis only included patients who did not receive a transfusion in the 31–365 days post-index period and had at least one lab value in the baseline period and in the period of interest. ^a^ Patients were only evaluated for a Hb response during six months if they had a Hb lab value recorded in the 31–180 days post-index. ^b^ Patients were only evaluated for a Hb response during 12 months if they had a Hb lab value recorded in the 181–365 days post-index.

**Table 3:** Breakthrough hemolysis for RAV (overall) patients, RAV (prior ECU) and ECU-treated patients.

		Patients with BTH		Person-Time (months) ^a^	
PNH Expert Consensus Definitions of BTH [24]	N ^b^	n	% [N/n]	Mean	SD
BTH defined as symptoms ±7 days ^c^ from elevated LDH ^d^					
RAV (overall) ^e^					
BTH after 6 months of treatment	32	1	3.1%	10.6	6.4
BTH after 12 months of treatment	23	1	4.3%	7.1	4.8
RAV (prior ECU)					
BTH 6 months after switch	24	1	4.2%	11.6	6.6
BTH 12 months after switch	18	1	5.6%	8.1	4.8
ECU					
BTH after 6 months of treatment	55	14	25.5%	28.4	31.8
BTH after 12 months of treatment	41	10	24.4%	30.6	31.6
BTH defined only as elevated LDH ^f^					
RAV (overall) ^e^					
BTH after 6 months of treatment	32	5	15.6%	9.8	6.7
BTH after 12 months of treatment	23	3	13.0%	6.6	5.0
RAV (prior ECU)					
BTH 6 months after switch	24	5	20.8%	10.5	7.2
BTH 12 months after switch	18	3	16.7%	7.4	5.3
ECU					
BTH after 6 months of treatment	55	34	61.8%	23.2	30.2
BTH after 12 months of treatment	41	24	58.5%	26.6	29.6
BTH defined as elevated LDH and decrease in Hb ^g^					
RAV (overall) ^e^					
BTH after 4 months of treatment	32	3	9.4%	11.3	7.0
RAV (prior ECU)					
BTH 4 months after switch	20	2	10.0%	13.3	6.7
ECU					
BTH after 4 months of treatment	44	12	27.3%	32.0	32.9

Abbreviations: BTH, breakthrough hemolysis; ECU, eculizumab; Hb, hemoglobin; LDH lactate dehydrogenase; RAV, ravulizumab. ^a^ Person-time defined as time between four, six, or 12 months after index date through first BTH event or, if no BTH event, the last C5i treatment plus 14 days for ECU or 56 days for RAV depending on patients’ last treatment. ^b^ Total patient Ns are different between treatment periods due to differences in data availability at four, six, and 12 months; time adjustments and dropouts are accounted for by reporting person time. ^c^ The same results were obtained when using symptoms ±1, three, or seven days from an elevated LDH. ^d^ BTH defined as LDH of at least 480 U/L (≥2 × ULN) and at least one new symptom or sign of intravascular hemolysis (i.e., fatigue, hemoglobinuria, abdominal pain, dyspnea, anemia (Hb < 10 g/dL), major adverse vascular event (including thrombosis), dysphagia, or erectile dysfunction) within one, three, or seven days of the elevated LDH (definition 1). ^e^ The RAV (overall) group included both RAV (ECU naïve) cohort and RAV (prior ECU) cohort. ^f^ BTH defined as LDH ≥ 2 × upper limit of normal (480 U/L) regardless of signs and symptoms (definition 2). ^g^ BTH defined as a 50% increase in LDH from baseline and Hb decrease of ≥2 g/dL from baseline; both measured at least four months after index; LDH and Hb records must have occurred within one week of one another (definition 3).

**Table 4:** Complement-amplifying conditions (CACs) proximal to breakthrough hemolysis (BTH) among RAV and ECU-treated patients with BTH.

	Ravulizumab-Treated Patients ^a^ N = 76					Eculizumab-Treated Patients N = 143				
	6-Month BTH		12-Month BTH		4-Month BTH	6-Month BTH		12-Month BTH		4-Month BTH
Number of patients eligible for BTH assessment	N = 32		N = 23		N = 32	N = 55		N = 41		N = 44
	Symptoms ± 7 days from elevated LDH ^b^	Elevated LDH ^c^	Symptoms ± 7 days from elevated LDH ^b^	Elevated LDH ^c^	Elevated LDH and Hb decrease ^d^	Symptoms ± 7 days from elevated LDH ^b^	Elevated LDH ^c^	Symptoms ± 7 days from elevated LDH ^b^	Elevated LDH ^c^	Elevated LDH and Hb decrease ^d^
Number of patients with BTH	*n* = 1	*n* = 5	*n* = 1	*n* = 3	*n* = 3	*n* = 14	*n* = 34	*n* = 10	*n* = 24	*n* = 12
Average person-time among those experiencing BTH (months) ^e^, mean SD	7.3 (-)	7.0 (1.0)	13.0 (-)	12.8 (0.7)	8.2 (3.4)	20.3 (19.4)	19.4 (20.0)	26.1 (20.4)	28.6 (19.7)	17.7 (12.8)
Any CAC within 15 days of BTH ^f^, n (%)	1 (100.0%)	3 (60.0%)	1 (100.0%)	1 (33.3%)	2 (66.7%)	11 (78.6%)	21 (61.8%)	6 (60.0%)	13 (54.2%)	8 (66.7%)
Infection ^g^	1 (100.0%)	2 (40.0%)	0 (0.0%)	0 (0.0%)	1 (33.3%)	6 (42.9%)	10 (29.4%)	4 (40.0%)	8 (33.3%)	4 (33.3%)
Upper respiratory tract infection	1 (100.0%)	2 (40.0%)	0 (0.0%)	0 (0.0%)	0 (0.0%)	3 (21.4%)	3 (8.8%)	2 (20.0%)	3 (12.5%)	1 (8.3%)
Surgery ^h^	1 (100.0%)	2 (40.0%)	1 (100.0%)	1 (33.3%)	2 (66.7%)	9 (64.3%)	16 (47.1%)	4 (40.0%)	9 (37.5%)	6 (50.0%)
Trauma ^i^	0 (0.0%)	0 (0.0%)	0 (0.0%)	0 (0.0%)	0 (0.0%)	1 (7.1%)	0 (0.0%)	1 (10.0%)	0 (0.0%)	0 (0.0%)
Pregnancy ^j^	0 (0.0%)	0 (0.0%)	0 (0.0%)	0 (0.0%)	0 (0.0%)	0 (0.0%)	1 (2.9%)	0 (0.0%)	1 (4.2%)	0 (0.0%)
Organ or hematopoietic stem cell transplantation ^k^	0 (0.0%)	0 (0.0%)	0 (0.0%)	0 (0.0%)	0 (0.0%)	1 (7.1%)	2 (5.9%)	1 (10.0%)	1 (4.2%)	0 (0.0%)
Acute transfusion reaction ^l^	0 (0.0%)	0 (0.0%)	0 (0.0%)	0 (0.0%)	0 (0.0%)	2 (14.3%)	1 (2.9%)	1 (10.0%)	0 (0.0%)	1 (8.3%)

Abbreviations: BTH, breakthrough hemolysis; CAC, complement-amplifying conditions; Hb, hemoglobin; LDH lactate dehydrogenase. ^a^ The RAV (overall) group included both RAV (ECU naïve) cohort and RAV (prior ECU) cohort. ^b^ BTH defined as LDH ≥ 2 × upper limit of normal (480 U/L) and at least one new symptom or sign of intravascular hemolysis (i.e., fatigue, hemoglobinuria, abdominal pain, dyspnea, anemia (Hb <10 g/dL), major adverse vascular event (including thrombosis), dysphagia, or erectile dysfunction) within one, three, or seven days of elevated LDH (definition 1). ^c^ BTH defined as LDH ≥ 2 × upper limit of normal (480 U/L) regardless of signs and symptoms (definition 2). ^d^ BTH defined as a 50% increase in LDH from baseline and Hb decrease of ≥2 g/dL from baseline; both measured at least four months after index; LDH and Hb records must have occurred within one week of one another (definition 3). ^e^ Person-time defined as time from index date through first BTH event or, if no BTH event, the last C5i treatment plus 14 days for ECU or 56 days for RAV depending on patients’ last treatment. ^f^ CAC is defined as proximal to BTH if it occurred within the 15 days before or after patients’ first BTH event in the relevant time frame. Patients may have had one or more CAC. A list of ICD-9 and ICD-10 codes used in the analyses is seen in Table S2. ^g^ Infection defined as any meningococcal infection, sepsis, aspergillus infection, viral infection, respiratory tract infection, urinary tract infection, cellulitis, pneumonia, or viral gastroenteritis. ^h^ Surgery defined as any invasive surgery, excluding events such as infusions or biopsies. ^i^ Trauma defined as any diagnosis of an injury to the body. ^j^ Pregnancy defined as any diagnosis or elevated chorionic gonadotropin. ^k^ Organ or hematopoietic stem cell transplantation defined as any procedure record of these events. ^l^ Acute transfusion reaction defined as a diagnosis of acute transfusion reaction.

**Table 5:** Incidence of long-term clinical outcomes in RAV and ECU-treated patients.

	RAV-Treated Patients ^a^ N = 76			ECU-Treated Patients N = 143		
	≥1 Event 30 Days after Index ^b^		≥2 Events 30 Days after Index ^b,c^	≥1 Event 30 Days after Index ^b^		≥2 Events 30 Days after Index ^b,c^
	n (%)	Person-Time, Months (SD)	n (%)	n (%)	Person-Time, Months (SD)	n (%)
Thrombosis ^d^	10 (13.2%)	11.9 (9.0)	7 (70.0%)	22 (15.4%)	25.9 (28.0)	17 (77.3%)
Cardiovascular ^e^	2 (2.6%)	14.0 (8.8)	1 (50.0%)	4 (2.8%)	28.2 (29.6)	3 (75.0%)
Persistent anemia ^f^	17 (22.4%)	11.2 (8.7)	17 (100%)	38 (26.6%)	25.5 (27.7)	38 (100%)
Kidney disease ^g^	7 (9.2%)	13.1 (9.0)	5 (71.4%)	27 (18.9%)	20.7 (27.4)	19 (70.4%)
Kidney injury ^h^	2 (2.6%)	13.9 (8.7)	1 (50.0%)	15 (10.5%)	25.6 (29.3)	8 (53.3%)
Iron overload ^i^	4 (5.3%)	13.3 (8.7)	4 (100.0%)	8 (5.6%)	28.4 (29.6)	8 (100.0%)
Infection ^j^	15 (19.7%)	10.8 (8.9)	6 (40.0%)	35 (24.5%)	22.1 (25.6)	23 (65.7%)
All-cause mortality ^k^	1 (1.3%)	8.6 (-)	--	10 (7.0%)	15.1 (19.3)	--
Infusion reaction ^l^	17 (22.4%)	11.1 (8.7)	5 (29.4%)	34 (23.8%)	23.3 (25.8)	26 (76.5%)

Abbreviations: C5i, C5 inhibitor; ECU, eculizumab; RAV, ravulizumab; SD, standard deviation. ^a^ The RAV (overall) group included both RAV (ECU naïve) cohort and RAV (prior ECU) cohort. ^b^ Patients who experienced outcome event at least 30 days after index date (i.e., treatment initiation); person-time for treated patients defined as time between 30 days after index date to first outcome event or, if no outcome event was recorded, date of last EMR record. ^c^ Patients who experienced at least two instances of outcome event at least 30 days apart. ^d^ Thrombosis defined as venous or arterial thrombosis or embolism. ^e^ Cardiovascular defined as acute myocardial infarction (MI) or congestive heart failure (CHF). ^f^ Persistent anemia defined as two instances of Hb < 10 g/dl at least eight weeks apart. ^g^ Kidney disease defined as diagnosis of chronic kidney disease (CKD), kidney failure, dependence on renal dialysis, or nephritis. ^h^ Kidney injury defined as acute kidney injury event or both eGFR < 60 and UACR > 30 mg/g. ^i^ Iron overload defined as a diagnosis of hemochromatosis. ^j^ Infection defined as any meningococcal infection, sepsis, aspergillus infection, viral infection, respiratory tract infection, urinary tract infection, cellulitis, pneumonia, or viral gastroenteritis. ^k^ All-cause mortality is available as reported by healthcare organizations (typically while inpatient) or as recorded in death registries. Date of death is obfuscated to the first day of the next month. ^l^ Infusion reaction defined as a diagnosis of angina, trouble breathing, shortness of breath, swelling, dizziness, anaphylaxis, or hypersensitivity reaction.

## Data Availability

The data used in this study were collected on 20 August 2021 from the TriNetX Dataworks Network, which provided access to electronic medical records (diagnoses, procedures, medications, laboratory values, genomic information) from approximately 55 million patients from 44 healthcare organizations who provide their data for use. TriNetX, LLC is compliant with the Health Insurance Portability and Accountability Act (HIPAA), the US federal law which protects the privacy and security of healthcare data, and any additional data privacy regulations applicable to the contributing healthcare organizations. TriNetX is certified to the ISO 27001:2013 standard and maintains an Information Security Management System (ISMS) to ensure the protection of the healthcare data it has access to and to meet the requirements of the HIPAA Security Rule. Any data displayed on the TriNetX Platform in aggregate form, or any patient level data provided in a data set generated by the TriNetX Platform, only contain de-identified data as per the de-identification standard defined in Section §164.514 (a) of the HIPAA Privacy Rule. The process by which the data are de-identified is attested to through a formal determination by a qualified expert as defined in Section §164.514(b)(1) of the HIPAA Privacy Rule.

## Supplementary Materials

The following supporting information can be downloaded at: https://www.mdpi.com/article/10.3390/hematolrep15020027/s1. TriNetX data source. Table S1. Distribution of the number of laboratory measures. Abbreviations: ARC, absolute reticulocyte count; ECU, eculizumab; Hb, hemoglobin; IQR, interquartile range; LDH, lactate dehydrogenase, LL, lower limit; RAV, ravulizumab; SD, standard deviation; UL, upper limit. ^a^ Number of unique patients. Figure S1. Study design (A) and example of timeframes for patients who switched (B). Figure S2. Mean (±SD) absolute reticulocyte count (ARC) from baseline through 12 months following initiation of C5 inhibitor (C5i) treatment. Abbreviations: ECU, eculizumab; IQR LL, interquartile range lower limit; IQR UL, interquartile range upper limit; RAV, ravulizumab; SD, standard deviation; ULN, upper limit of normal. Note: 1.5 times the ULN for ARC was defined as 120 × 10^9^/L. Table S2: List of codes and variable definitions used in the study.
