# Supplementary material for: Changes in Hematologic Lab Measures Observed in Patients with Paroxysmal Nocturnal Hemoglobinuria Treated with C5 Inhibitors, Ravulizumab and Eculizumab: Real-World Evidence from a US Based EMR Network
Source: Hematol Rep. 2023 Apr 21;15(2):266–82. doi: 10.3390/hematolrep15020027 (PMC10123631; doi:10.3390/hematolrep15020027)
Supplement: Supplementary file 1 [file hematolrep-15-00027-s001.zip › hematolrep-2226278-supplementary.pdf]

Supplemental Materials

Title: Changes in Hematologic Lab Measures Observed in Patients with Paroxysmal Nocturnal Hemoglobinuria Treated with C5 Inhibitors, Ravulizumab and Eculizumab: Real-World Evidence from a US Based EMR Network

Author: Jesse Fishman[1*], Seth Kuranz[2], Michael M Yeh[1], Kaylen Brzozowski[2], Herman Chen[2]

Affiliations:

[1]Apellis Pharmaceuticals, Inc., Waltham, MA 02451, USA

[2]TriNetX, LLC, Cambridge, MA 02140, USA

Contents

TriNetX data source 3

Table S1. Distribution of the number of laboratory measures 5

Figure S1. Study design (A) and example of timeframes for patients who switched (B) 6

Figure S2. Mean (±SD) absolute reticulocyte count (ARC) from baseline through 12 months following initiation of C5 inhibitor (C5i) treatment. 7

Table S2: List of codes and variable definitions used in the study 8

TriNetX data source

TriNetX, LLC ("TriNetX") is a global federated electronic medical record (EMR) network. Network members include academic medical centers, integrated delivery networks, specialty hospitals, and large specialty physician practices. The Dataworks-USA Network is comprised of inpatient and outpatient EMRs from 44 US member HCOs that allow the data to be used for research purposes as long as the contributor is not identified and data are pooled across the contributing HCOs.

TriNetX maps the data to a standard and controlled set of clinical terminologies. The data is then transformed into a proprietary data schema. This transformation process includes an extensive data quality assessment that includes 'data cleaning' that rejects records that do not meet the TriNetX quality standards. The data elements in TriNetX include, but are not limited to:

- **Demographic data** include elements such as age, gender, race, ethnicity, marital status, and US Census region.
- **Encounter data** include elements such as start date, end date, and encounter type (ambulatory, emergency room, inpatient, home health, inpatient non-acute, observation, pre-admission, short stay, and virtual).
- **Diagnosis data** are mapped to ICD-10-CM and include diagnosis code, date, and whether the diagnosis was indicated as primary, secondary, or unknown. Diagnoses may also be indicated as admitting diagnoses or reason for visit.
- **Procedure data** are mapped to ICD-10-PCS, CPT, SNOMED, or HCPCS and include code, date, and whether the procedure was indicated as primary, secondary, or unknown.

- **Medication data** are mapped by ingredient to RxNorm and include code, start date, route of administration, brand, strength, quantity dispensed, and days supply.
- **Lab data** are mapped to LOINC or TriNetX custom codes and include code, date recorded, lab result (numeric or positive/negative/unknown), and units of measure.
- **Vital Signs** are mapped to LOINC or TriNetX custom codes and include code, date, value, and units of measure

A detailed description of the TriNetX data source can be found in the Supplementary Material of "Taquet M, Geddes JR, Husain M, Luciano S, Harrison PJ. 6-month neurological and psychiatric outcomes in 236 379 survivors of COVID-19: a retrospective cohort study using electronic health records. *Lancet Psychiatry*. 2021;8(5):416-427. doi:10.1016/S2215-0366(21)00084-5"

More information on TriNetX and the data quality process can be found in "Topaloglu U, Palchuk MB. Using a federated network of real-world data to optimize clinical trials operations. JCO Clin Cancer Inform. 2018;2:1-10. doi:10.1200/CCI.17.00067"

Table S1. Distribution of the number of laboratory measures

RAV (Prior ECU)						
Total labs	n[a]	Mean	SD	Median	LL IQR	UL IQR
Baseline						
Hb	39	21.1	28.9	14	3	25
LDH	32	10.6	10.7	6	2	18
ARC	19	6.1	8.7	2	1	6
1-90 days post-index						
Hb	28	4.9	6.1	2.5	1	7.5
LDH	21	2.3	1.6	2	1	3
ARC	5	2.2	1.3	2	1	3
91-180 days post-index						
Hb	27	3.4	3.8	2	1	4
LDH	20	1.9	1.5	1	1	2
ARC	7	1.6	0.8	1	1	2
181-365 days post-index						
Hb	25	4.8	2.5	4	3	5
LDH	19	3.2	1.4	3	2	4
ARC	7	1.7	1.3	1	1	3

RAV (ECU Naïve)						
Total labs	n[a]	Mean	SD	Median	LL IQR	UL IQR
Baseline						
Hb	29	10.7	16.2	5	1	13
LDH	26	4.7	5.8	3	1	5
ARC	11	2.0	1.1	2	1	3
1-90 days post-index						
Hb	16	4.2	4.4	2	1	5.5
LDH	13	2.3	1.9	2	1	2
ARC	4	1.8	1.5	1	1	2.5
91-180 days post-index						
Hb	14	3.9	5.7	2	1	3
LDH	13	2.0	1.5	1	1	2
ARC	2	2.5	2.1	2.5	1	4
181-365 days post-index						
Hb	10	8.8	11.0	4	3	11
LDH	8	4.9	3.4	4	2.5	7
ARC	3	2.7	2.9	1	1	6

ECU						
Total labs	n[a]	Mean	SD	Median	LL IQR	UL IQR
Baseline						
Hb	126	16.3	21.0	9	3	22
LDH	89	7.3	11.2	4	2	8
ARC	43	3.9	5.5	2	1	3
1-90 days post-index						
Hb	93	11.0	14.6	6	4	10
LDH	67	5.4	3.7	5	2	8
ARC	19	3.7	3.4	4	1	5
91-180 days post-index						
Hb	66	7.0	6.6	5	3	8
LDH	48	5.4	4.7	4	3	6
ARC	11	6.4	6.2	5	2	8
181-365 days post-index						
Hb	65	13.5	15.2	10	4	15
LDH	49	9.1	10.8	5	2	13
ARC	16	4.1	4.3	2	1.5	5

Abbreviations: ARC, absolute reticulocyte count; ECU, eculizumab; Hb, hemoglobin; IQR, interquartile range; LDH, lactate dehydrogenase, LL, lower limit; RAV, ravulizumab; SD, standard deviation; UL, upper limit.
[a]Number of unique patients

Figure S1. Study design (A) and example of timeframes for patients who switched (B)

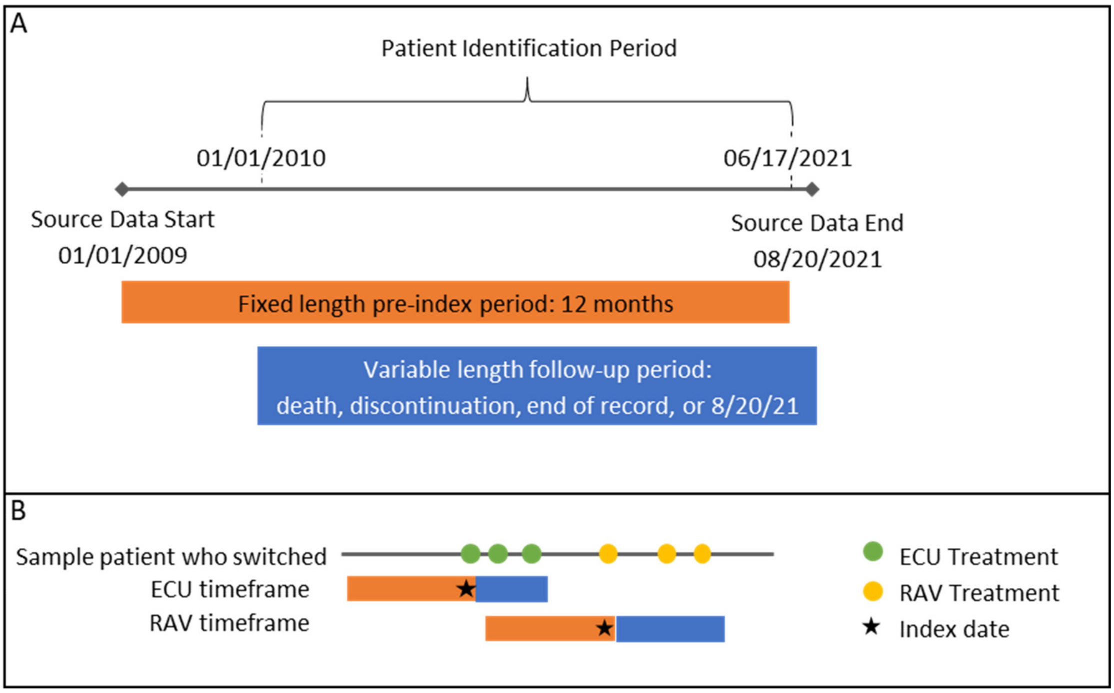

Figure S2. Mean (±SD) absolute reticulocyte count (ARC) from baseline through 12 months following initiation of C5 inhibitor (C5i) treatment.

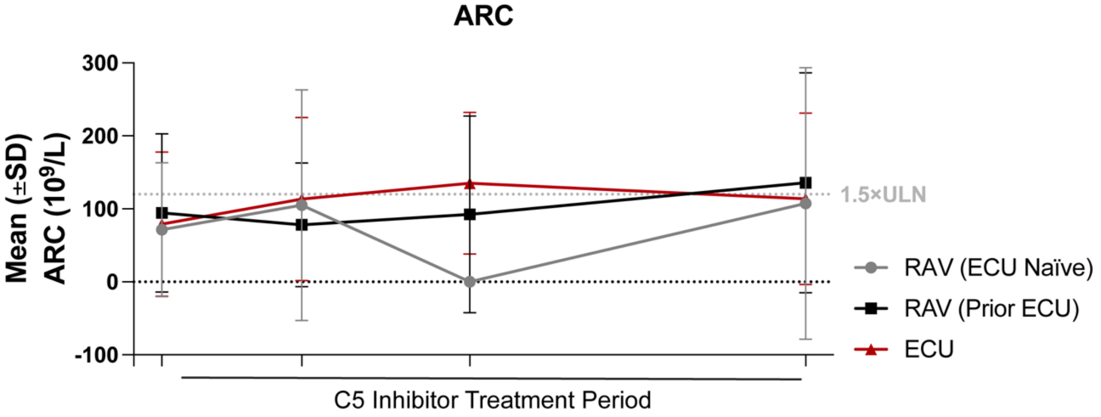

	Baseline	1–3 Months	4–6 Months	7–12 Months
**RAV (ECU Naïve)**				
N	11	4	2	3
Mean	71.4	105.2	0.2	107.5
SD	91.8	158.2	0.2	186.0
Median	54.0	43.1	0.2	0.1
IQR LL	0.1	0.1	0.1	0.1
IQR UL	141.4	210.3	0.3	322.2
**RAV (Prior ECU)**				
N	19	5	7	7
Mean	94.5	78.0	92.4	135.9
SD	108.5	84.9	134.8	150.7
Median	66.6	60.4	0.2	67.1
IQR LL	0.1	0.2	0.1	0.2
IQR UL	164.0	138.2	254.4	293.0
**ECU**				
N	43	19	11	16
Mean	79.2	113.4	135.3	113.7
SD	98.8	111.9	97.3	117.5
Median	14.5	91.5	125.9	90.4
IQR LL	0.2	0.3	48.7	0.3
IQR UL	145.2	183.0	190.4	180.1

Abbreviations: ECU, eculizumab; IQR LL, interquartile range lower limit; IQR UL, interquartile range upper limit; RAV, ravulizumab; SD, standard deviation; ULN, upper limit of normal.
Note: 1.5 times the ULN for ARC was defined as 120×10^9/L.

Table S2: List of codes and variable definitions used in the study

TriNetX Ms_TableS2
